# Implante Percutâneo Transeptal de Bioprótese em Disfunção de Prótese Valvar Cirúrgica Mitral – Experiência Multicêntrica Brasileira

**DOI:** 10.36660/abc.20190252

**Published:** 2020-09-18

**Authors:** Pedro Felipe Gomes Nicz, Pedro Henrique M. Craveiro de Melo, Pedro Henrique Ferro de Brito, Eliane Nogueira Lima, Ricardo Cavalcante e Silva, Maurício Lopes Prudente, Fernando Henrique Fernandes, Maurilio Onofre Deininger, Marcelo Antônio Cartaxo Queiroga Lopes, Fúlvio Soares Petrucci, Fernando Roquette Reis, Marcos Antonio Marino, Rodrigo de Castro Bernardes, Eduardo Pessoa de Melo, Marco Antonio Praça Oliveira, José Armando Mangione, Fernanda Marinho Mangione, Carlos Henrique Eiras Falcão, Estêvão Carvalho de Campos Martins, Walter Lunardi, Fernando Bacal, Flávio Tarasoutchi, Fábio Sândoli de Brito

**Affiliations:** 1 Universidade de São Paulo Faculdade de Medicina Hospital das Clínicas São Paulo SP Brasil Universidade de São Paulo Faculdade de Medicina Hospital das Clínicas Instituto do Coração – Hemodinâmica, São Paulo, SP - Brasil; 2 Hospital São Camilo Pompeia São Paulo SP Brasil Hospital São Camilo Pompeia - Cardiologia Intervencionista, São Paulo, SP - Brasil; 3 Hospital Sírio-Libanês São Paulo SP Brasil Hospital Sírio-Libanês, São Paulo, SP - Brasil; 4 Faculdade de Ciências Médicas Santa Casa de São Paulo São Paulo SP Brasil Faculdade de Ciências Médicas da Santa Casa de São Paulo, São Paulo, SP - Brasil; 5 Hospital Encore Goiânia GO Brasil Hospital Encore, Goiânia, GO - Brasil; 6 Hospital Alberto Urquiza Wanderley João Pessoa PB Brasil Hospital Alberto Urquiza Wanderley, João Pessoa, PB - Brasil; 7 Hospital Memorial São Francisco João Pessoa PB Brasil Hospital Memorial São Francisco, João Pessoa, PB - Brasil; 8 Hospital Madre Teresa Belo Horizonte MG Brasil Hospital Madre Teresa, Belo Horizonte, MG - Brasil; 9 Hospital Esperança Olinda Rede D’Or São Luiz Olinda PE Brasil Hospital Esperança Olinda - Rede D’Or São Luiz, Olinda, PE - Brasil; 10 Hospital Beneficência Portuguesa de São Paulo São Paulo SP Brasil Hospital Beneficência Portuguesa de São Paulo, São Paulo, SP- Brasil; 11 Complexo Hospitalar de Niterói Niterói Brasil Complexo Hospitalar de Niterói, Niterói, RJ - Brasil; 12 Hospital de Força Aérea do Galeão Rio de Janeiro RJ Brasil Hospital de Força Aérea do Galeão, Rio de Janeiro, RJ - Brasil; 13 Universidade de São Paulo Instituto do Coração São Paulo SP Brasil Universidade de São Paulo Instituto do Coração, São Paulo, SP - Brasil; 14 Universidade de São Paulo Faculdade de Medicina Hospital das Clínicas São Paulo SP Brasil Universidade de São Paulo Faculdade de Medicina Hospital das Clínicas Instituto do Coração – Cardiologia, São Paulo, SP – Brasil

**Keywords:** Estenose da Valva Mitral/cirurgia, Substituição da Valva Mitral Transcateter, Bioprótese, Ecocardiografia Transesofagiana/métodos, Implante de Prótese de Valva Cardíaca/tendências

## Abstract

**Fundamento:**

A intervenção percutânea em pacientes com disfunção de prótese biológica mitral apresenta-se como uma alternativa ao tratamento cirúrgico convencional.

**Objetivo:**

Relatar a primeira experiência brasileira de implante transcateter de bioprótese *valve-in-valve* mitral via transeptal (TMVIV-via transeptal).

**Métodos:**

Foram incluídos pacientes portadores de disfunção de bioprótese cirúrgica submetidos ao TMVIV-transeptal em 12 hospitais brasileiros. Foram considerados estatisticamente significativos valores de p<0,05.

**Resultados:**

Entre junho/2016 e fevereiro/2019, 17 pacientes foram submetidos ao TMVIV-via transeptal. A mediana de idade foi 77 anos (IIQ,70-82), a mediana do escore STS-PROM foi 8,7% (IIQ,7,2-17,8). Todos os pacientes tinham sintomas limitantes de insuficiência cardíaca (CF≥III), tendo 5 (29,4%) sido submetidos a mais de uma toracotomia prévia. Obteve-se sucesso do TMVIV-via transeptal em todos os pacientes. A avaliação ecocardiográfica demonstrou redução significativa do gradiente médio (pré-intervenção, 12±3,8 mmHg; pós-intervenção, 5,3±2,6 mmHg; p<0,001), assim como aumento da área valvar mitral (pré-intervenção, 1,06±0,59 cm^2^; pós-intervenção, 2,18±0,36 cm^2^; p<0,001) sustentados em 30 dias. Houve redução significativa e imediata da pressão sistólica de artéria pulmonar, com redução adicional em 30 dias (pré-intervenção, 68,9±16,4 mmHg; pós-intervenção, 57,7±16,5 mmHg; 30 dias,50,9±18,7 mmHg; p<0,001). Durante o seguimento, com mediana de 162 dias (IIQ, 102-411), observou-se marcada melhora clínica (CF≤II) em 87,5%. Um paciente (5,9%) apresentou obstrução de via de saída de ventrículo esquerdo (VSVE), evoluindo para óbito logo após o procedimento, e outro morreu aos 161 dias de seguimento.

**Conclusão:**

A primeira experiência brasileira de TMVIV-transeptal demonstra a segurança e a efetividade dessa nova técnica. A obstrução da VSVE é uma complicação potencialmente fatal, reforçando a importância da seleção dos pacientes e do planejamento do procedimento. (Arq Bras Cardiol. 2020; 115(3):515-524)

## Introdução

A substituição cirúrgica da valva mitral e o reparo cirúrgico são o tratamento de escolha para grande parte dos acometimentos primários da valva mitral; entretanto, a degeneração da prótese e a consequente disfunção limitam a durabilidade da terapia a longo prazo.^1-3^ Até 35% dos pacientes submetidos ao tratamento cirúrgico da valva mitral necessitam de nova intervenção após uma mediana de 8 anos, com uma taxa de mortalidade intra-hospitalar variando entre 8% e 12% e tempo médio de hospitalização de 17 dias.^4-8^ Nesse contexto, muitos dos pacientes que cursam com disfunção de prótese mitral e indicação de intervenção cirúrgica, especialmente aqueles portadores de múltiplas comorbidades e manipulações cirúrgicas prévias, têm a cirurgia contraindicada devido ao alto risco associado ao procedimento. Nessas situações, a intervenção transcateter apresenta-se como uma alternativa ao tratamento cirúrgico.

Para a intervenção transcateter, a via transapical, já realizada inclusive em nosso meio, tem a vantagem do acesso direto à valva mitral pelo ápice cardíaco e de apresentar menor morbidade que a cirurgia convencional.^9,10^Entretanto, associa-se a elevadas taxas de complicações hemorrágicas, necessidade de drenagem torácica pela abertura da pleura e, consequentemente, tempo prolongado de hospitalização.^11-13^

Com o objetivo de reduzir o risco do procedimento e o tempo de hospitalização, desenvolveu-se a técnica de implante transcateter de bioprótese *valve-in-valve* em posição mitral por via transeptal (TMVIV-via transeptal). Relatos iniciais e séries de casos demostraram a viabilidade da técnica e a segurança do procedimento, com bons resultados clínicos no seguimento de médio prazo.^14^

Apresentamos a primeira experiência brasileira de TMVIV-via transeptal para o tratamento de pacientes portadores de disfunção de prótese biológica mitral e com alto risco para uma nova intervenção cirúrgica.

## Métodos

Pacientes portadores de disfunção significativa de bioprótese mitral cirúrgica (estenose, insuficiência ou ambas), submetidos ao TMVIV-via transeptal em 12 hospitais brasileiros, foram incluídos no presente estudo. Trata-se de um procedimento que, durante a realização dessa série de casos, não constava das indicações de bula do dispositivo empregado (situação *off-label*), apesar de amplamente realizado em diversos países do mundo. Mais recentemente, houve aprovação dessa indicação também no Brasil.

Todos os pacientes foram avaliados pelo *heart team* local, sendo classificados como de alto risco cirúrgico. Dados demográficos, clínicos, de exames complementares e do procedimento foram compilados. O seguimento clínico ocorreu de acordo com a prática médica local, assim como a indicação do uso de antiagregantes e/ou anticoagulantes após a intervenção

### Planejamento pré-procedimento

O ecocardiograma transesofágico pré-procedimento foi realizado em todos os casos para documentar e quantificar a disfunção valvar, assim como para avaliar o mecanismo da disfunção protética, a presença de regurgitação paravalvar e os sinais sugestivos de endocardite infecciosa.

**Figura 1:**
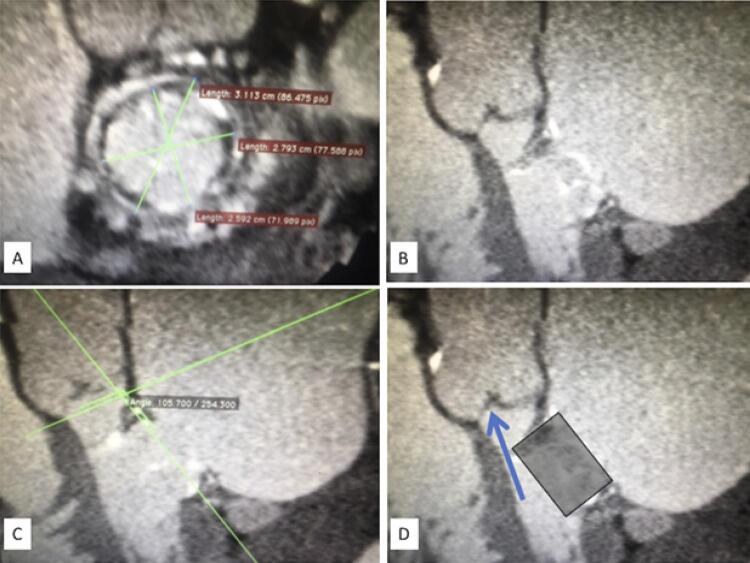
– Planejamento pré-procedimento - avaliação tomografia computadorizada cardíaca.15-17

A angiotomografia pré-procedimento permitiu avaliar o diâmetro interno verdadeiro da prótese disfuncional, sua angulação e a relação com a via de saída de ventrículo esquerdo (VSVE), como ilustra a Figura 1.^15-17^ Quando disponível, o relato da cirurgia prévia foi útil na obtenção de informação sobre o tipo e especificações técnicas das próteses implantadas.

### Procedimentos

Os procedimentos foram realizados em salas de hemodinâmica tradicionais ou em salas híbridas, com o paciente sob anestesia geral e auxílio de ecocardiograma transesofágico intraoperatório. A artéria femoral esquerda foi acessada com introdutor 5F para monitorização invasiva da pressão arterial e eventual ventriculografia esquerda. A veia femoral esquerda foi utilizada para posicionamento de cabo de marca-passo transvenoso provisório. A veia femoral direita foi utilizada para a punção transeptal e posterior implante da prótese transcateter. Um dispositivo Perclose ProGlide® (Abbott Vascular Devices, Santa Clara, Califórnia, EUA) foi pré-posicionado para posterior hemostasia venosa.

Durante o procedimento, o septo interatrial foi puncionado sob orientação ecocardiográfica, utilizando as técnicas habituais. Para permitir melhor manipulação dos dispositivos no átrio esquerdo e o cruzamento da valva mitral, optou-se por realizar a punção septal na região posterior e inferior ou média do septo.

Administrou-se heparina objetivando atingir um tempo de coagulação ativada acima de 300 segundos. Após a punção do septo, através de um guia 0,035” de troca na veia pulmonar superior esquerda, avançou-se um cateter Agilis NXT 8F (St Jude Medical, St Paul, MN, EUA) até o átrio esquerdo. Um cateter diagnóstico JR 5F foi empregado para ultrapassar a valva mitral utilizando-se um guia hidrofílico (ponta reta), guiado pelo ecocardiograma transesofágico e por fluoroscopia, em projeção perpendicular ao anel valvar mitral. Posicionou-se então um ou dois guias Safari (Boston Scientific, MN, EUA) ou Amplatz Extra-Stiff (Cook Medical, Bloomington, Indiana, EUA) no ventrículo esquerdo. No primeiro caso da série, optou-se pelo uso da técnica de *arterial loop*, laçando e exteriorizando o fio guia 0,035” pelo acesso arterial femoral para facilitar a navegação e o posicionamento da prótese.

Próteses expansíveis por balão SAPIEN XT e SAPIEN 3 (Edwards LifeSciences, Irvine, Califórnia, EUA) foram escolhidas devido a características, como baixo perfil, flexibilidade, força radial, experiência prévia em implantes *valve-in-valve*, bem como bom desempenho em relatos e séries de casos internacionais.^15,18-22^ A escolha do tamanho da prótese foi realizada utilizando a avaliação da angiotomografia e dos dados do fabricante da prótese cirúrgica, objetivando um sobredimensionamento de 5-10%. A prótese foi posicionada no cateter-balão no sentido utilizado para implante anterógrado, assim como é preparada para o implante transapical aórtico.

O introdutor específico da prótese SAPIEN 3 ou SAPIEN XT foi posicionado e o septo interatrial dilatado com a insuflação de balões de 12 a 16 mm de diâmetro por 40 mm de comprimento.

Após a dilatação do septo, introduziu-se a prótese em seu sistema de liberação na veia cava inferior, onde foi ajustada e alinhada ao balão. Auxiliada pelo sistema de flexão do dispositivo de liberação, a prótese foi avançada através do septo e posicionada em topografia mitral. Nesse momento, guiado pelo ecocardiograma transesofágico e pela fluoroscopia, posicionou-se o dispositivo no local ideal para a liberação. Com a prótese posicionada, utilizou-se o marca-passo em frequência de 180 bpm (*rapid pacing*) e insuflou-se lentamente o balão de liberação, realizando-se pequenos ajustes de posicionamento, conforme necessário, sempre procurando atingir a posição final da prótese 10-20% atrial e 80-90% ventricular, com a face ventricular mais expandida em relação à face atrial, para minimizar a possibilidade de embolização atrial do dispositivo.

**Figura 2:**
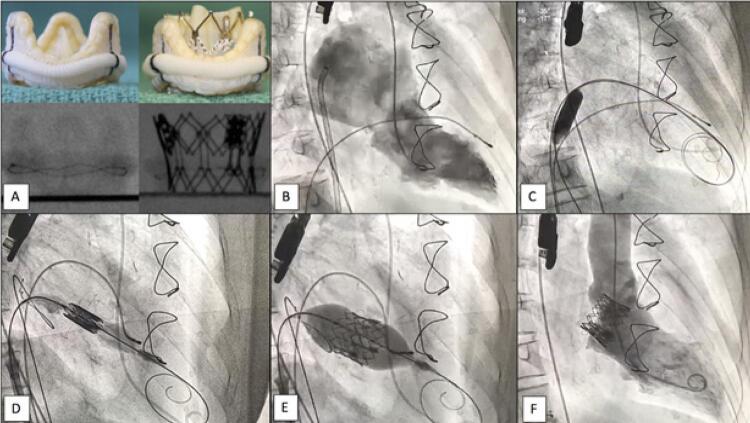
– Procedimento - implante transcateter de bioprótese cirúrgica valve-in-valve em posição mitral por via transeptal.

Após a liberação da prótese, uma avaliação detalhada do implante e do funcionamento da valva mitral foi realizada e repetida após a retirada do sistema de liberação das cavidades esquerdas. A avaliação e a quantificação da comunicação interatrial residual foram realizadas na sequência. Ao término do procedimento, realizou-se uma ventriculografia esquerda para avaliar o posicionamento, a presença de regurgitação mitral e possíveis complicações relacionadas ao procedimento. O efeito da heparina foi revertido e o introdutor femoral foi removido com auxílio de um dispositivo hemostático Perclose ProGlide® (Abbott Vascular Devices, Santa Clara, Califórnia, EUA) previamente posicionado. O introdutor arterial e o marca-passo provisório foram retirados logo após o procedimento. A Figura 2 ilustra os passos do procedimento.

### Análise estatística

Todas as variáveis contínuas foram testadas para normalidade, utilizando-se os testes de Kolmogorov–Smirnov ou Shapiro-Wilk, ambos corroborando os resultados. As variáveis contínuas com distribuição normal foram apresentadas como média e desvio-padrão e as sem distribuição normal, como mediana e intervalo interquartil (IIQ). As variáveis categóricas foram apresentadas como frequências (número e porcentagem). Para a análise sequencial das variáveis contínuas no mesmo paciente, utilizou-se o método de equações de estimação generalizadas (GEE) com comparações múltiplas de Bonferroni. Para a análise da evolução da classe funcional (CF) de insuficiência cardíaca, utilizou-se o teste de Wilcoxon pareado. O *software* utilizado para realizar as análises estatísticas foi o SPSS para Windows, versão 22.0. Os valores de p<0,05 foram considerados estatisticamente significativos.

### Considerações éticas

Os termos de consentimento relacionados ao procedimento foram aplicados antes de cada intervenção. O presente estudo foi submetido ao Comitê de Ética em Pesquisa e por ele aprovado, tendo sido realizado de acordo com a Resolução 466/12 e suas complementares do Conselho Nacional de Saúde.

## Resultados

Entre junho de 2016 e janeiro de 2019, 17 pacientes foram submetidos ao TMVIV-via transeptal sempre com a presença de um operador experiente e familiarizado com a técnica (FSBJ). As características demográficas e clínicas basais estão apresentadas na Tabela 1. A mediana de idade dos pacientes foi 77 anos (IIQ, 70-82), variando entre 29 e 85 anos, sendo 11 pacientes (64,7%) do sexo feminino e 9 (52,9%) portadores de fibrilação atrial.

**Tabela 1:** – Características basais

	(n = 17)
Idade, anos	73,8 ± 13,0
Sexo feminino, n (%)	11 (64,7)
NYHA classe III ou IV, n (%)	17 (100)
NYHA classe IV, n (%)	9 (52,9)
STS escore, %	16,2 ± 16,0
Cirurgias cardíacas prévias (≥ 2 cirurgias), n (%)	5 (29,4)
Diabetes mellitus, n (%)	5 (29,4)
Creatinina, mg/dl	1,3 ± 0,7
Hipertensão, n (%)	8 (47,0)
Fibrilação atrial, n (%)	9 (52,9)
AVC prévio, n(%)	4 (23,5)
Doença pulmonar crônica, n (%)	9 (52,9)
IAM prévio, n (%)	4 (23,5)
Revascularização cirúrgica prévia, n (%)	9 (52,9)
Angioplastia coronária prévia, n (%)	3 (17,6)
Dados ecocardiográficos	
Gradiente médio, mmHg	10,6 ± 5,4
PSAP, mmHg	68,9 ±16,4
FEVE, %	59,3 ± 13,0
Mecanismo de disfunção	
Insuficiência, n (%)	3 (17,6)
Estenose, n (%)	10 (58,8)
Combinado, n (%)	4 (23,5)

*NYHA: New York Heart Association; STS: Society of Thoracic Surgeons; AVC: acidente vascular cerebral; IAM: infarto agudo do miocárdio; PSAP: pressão sistólica da artéria pulmonar; FEVE: fração de ejecção d ventrículo esquerdo.*

As etiologias primárias da disfunção valvar mitral mais frequentes foram a degeneração mixomatosa e o acometimento reumático, ambos ocorrendo em 7 casos (41,2%). A mediana de escore de risco STS (*Society of Thoracic Surgeons*) foi 8,7% (IIQ, 7,2-17,8%). Cinco pacientes (29,4%) tinham mais de uma cirurgia cardíaca prévia e a mediana de tempo desde a última troca valvar mitral foi de 9 anos (IIQ, 8-10). Todos os pacientes encontravam-se com limitação funcional importante, em CF III ou IV, e significativa hipertensão arterial pulmonar, com mediana de pressão sistólica de artéria pulmonar (PSAP) de 69,5 mmHg (IIQ, 57,3-73). A mediana de fração de ejeção de ventrículo esquerdo (FEVE) foi de 63% (IIQ, 57-65%), sendo que 2 pacientes apresentavam disfunção ventricular esquerda importante com FEVE menor que 35%.

O mecanismo de disfunção mais comum foi a estenose protética mitral pura, presente em 10 (58,8%) pacientes, outros 4 (23,5%) apresentavam disfunção combinada e 3 (17,6%) apresentavam insuficiência mitral pura, como ilustra a Tabela 2. A avaliação angiotomográfica foi realizada em 15 (88,2%) pacientes e indicou que a mediana do ângulo entre os planos mitral e aórtico foi de 123^O^ (IIQ, 117-134), sendo que, em 35,3% dos casos, o ângulo era inferior a 120^O^.

**Tabela 2:** – Características hemodinâmicas e valvares por paciente

Paciente #	Prótese prévia	Idade da prótese (anos)	Tipo de disfunção	Tamanho (mm)	SAPIEN modelo	SAPIEN tamanho (mm)	Gradiente médio (mmHg)		Grau de regurgitação		PSAP (mmHg)	
							pré	pós	pré	pós	pré	pós (30 dias)
1	Biocardio	8	Estenose	27	XT	26	18	8	0	0	90	87
2	St. Jude Biocor	3	Estenose	29	XT	26	16	12	0	0	82	72
3	Braile	8	Estenose	31	S3	29	12	-	1	0	69	-
4	Indeterminado	3	Combinada	29	S3	29	7	3	4	0	65	-
5	Indeterminado	10	Combinada	29	XT	26	9	6	4	1	105	75
6	St. Jude Biocor	12	Estenose	29	XT	26	10	4	0	0	87	-
7	St. Jude Biocor	14	Insuficiência	31	XT	29	-	9	4	0	53	40
8	Indeterminado	6	Estenose	29	XT	26	13	3	0	1	-	30
9	Indeterminado	13	Estenose	25	S3	26	20	5	1	0	70	-
10	Carpentier-Edwards	9	Estenose	29	S3	29	14	4	1	0	55	38
11	Carpentier-Edwards	8	Estenose	29	S3	29	12	5	1	0	68	51
12	Carpentier-Edwards Magna	12	Combinada	29	S3	29	10	4	4	0	70	57
13	Carpentier-Edwards	10	Estenose	29	S3	29	8	3	1	0	40	36
14	Carpentier-Edwards Magna	10	Estenose	29	S3	29	13	3	1	0	50	55
15	Braile	10	Combinada	27	S3	26	11	6	2	1	58	38
16	Labcor	8	Insuficiência	31	S3	29	-	4	4	0	70	-
17	Cardioprotese	1	Insuficiência	29	S3	26	7	-	4	0	70	32

*PSAP: pressão sistólica da artéria pulmonar.*

O posicionamento e o implante foram realizados conforme o planejado, por via transeptal, em todos os casos. Em cinco casos, a prótese mitral disfuncional não apresentava nenhuma marca radiopaca que auxiliasse o implante valvar percutâneo, sendo que nesses pacientes o posicionamento da prótese transcateter foi guiado por ecocardiograma transesofágico tridimensional.

As medianas de tempos de procedimento e de fluoroscopia e de volume de contraste foram 125 minutos (IIQ, 100-148), 25 minutos (IIQ, 22-40) e 50 ml (IIQ, 43-141ml), respectivamente. Foram utilizadas próteses transcateter, balão expansível, SAPIEN XT em 6 (35,3%) casos e SAPIEN 3 em 11 (64,7%). A prótese de tamanho 29 mm foi a escolhida em 52,9% dos casos, tendo-se optado pela de 26 mm (47,1%) nos demais, conforme aponta a Tabela 2.

Um paciente (5,9%), apesar do posicionamento e da liberação da prótese conforme planejado, evoluiu com obstrução de VSVE, detectada pelo ecocardiograma e confirmada com medida direta do gradiente intraventricular, seguida de rápida deterioração hemodinâmica, choque cardiogênico refratário e óbito horas após o procedimento. Nesse paciente, pela extrema gravidade clínica basal, não foi possível realizar a angiotomografia prévia à intervenção. Em outro caso, detectou-se gradiente invasivo na VSVE de 20 mmHg logo após o procedimento, sem repercussão clínica. Não ocorreram outras complicações relacionadas ao procedimento, como necessidade de conversão para cirurgia de emergência, infarto agudo do miocárdio, acidente vascular cerebral (AVC), complicações vasculares maiores e sangramentos maiores. No período de 30 dias, nenhum paciente apresentou trombose de prótese ou AVC ou necessitou de novas intervenções cardíacas. A mediana do tempo de internação foi 7 dias (IIQ, 4-14).

**Figura 3:**
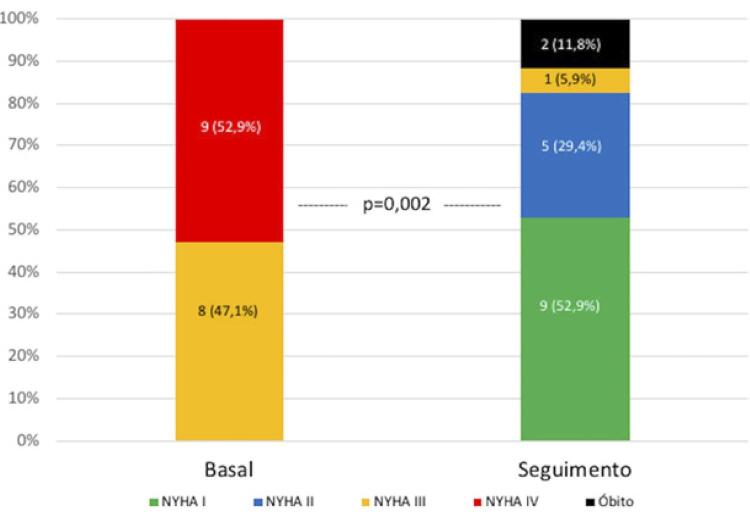
– Classe funcional - basal vs seguimento.

Durante o seguimento, com mediana de 162 dias (IIQ, 102-411), os pacientes da presente série apresentaram evolução clínica favorável, com melhora marcada da CF (≤ II) em 14 pacientes (87,5%), como demonstra a Figura 3. Reinternações por insuficiência cardíaca ocorreram em 4/16 pacientes (25%) que receberam alta hospitalar. Um paciente, que durante o acompanhamento permaneceu em CF IV, com hipertensão pulmonar severa e múltiplas reinternações, evoluiu para óbito 161 dias após a intervenção. A taxa de sucesso do procedimento, segundo as definições estabelecidas através do documento redigido pelo *Mitral Valve Academic Research Consortium* (MVARC),^23^ foi de 88,2%.

**Figura 4:**
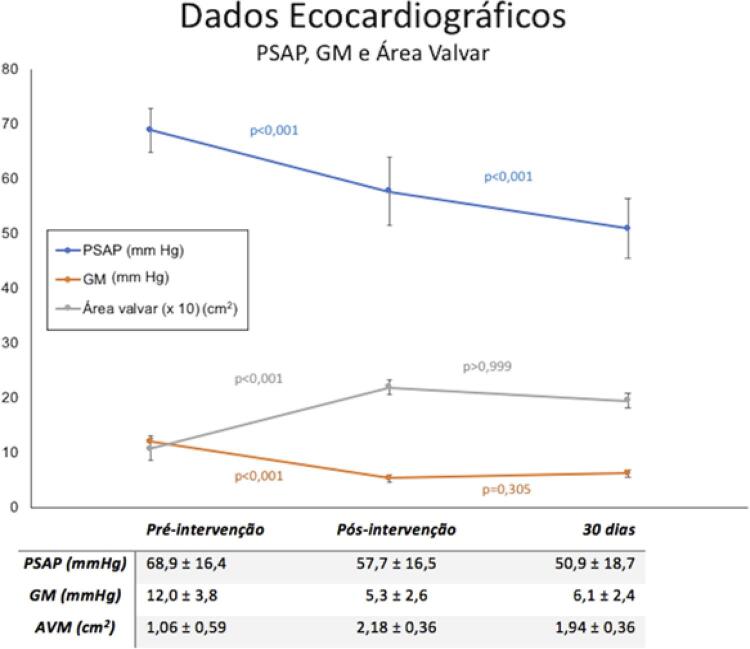
– Dados ecocardiográficos pré-intervenção, pós-intervenção e com 30 dias de seguimento. PSAP: pressão sistólica da artéria pulmonar; GM: gradiente mitral; AVM: área da valva mitral

A avaliação ecocardiográfica demonstrou redução significativa do gradiente médio transvalvar mitral, sustentada em 30 dias e associada ao aumento da área valvar (Figura 4). Houve, ainda, redução significativa da PSAP, que ocorreu imediatamente após o procedimento, com redução adicional após 30 dias de acompanhamento (Figura 4). Nenhuma regurgitação mitral maior que discreta (>1+) foi detectada após os implantes, assim como nenhum dos casos apresentou comunicação interatrial hemodinamicamente significativa com necessidade de intervenção imediata ou durante o acompanhamento.

## Discussão

Os dados apresentados nesta série a respeito da experiência inicial com o TMVIV-via transeptal em disfunção de prótese mitral cirúrgica demonstram que esse procedimento é seguro e eficaz, com resultados semelhantes àqueles observados em séries internacionais, devendo ser considerado como uma alternativa ao tratamento cirúrgico tradicional em pacientes de alto risco.

A abordagem percutânea da disfunção de próteses biológicas aórticas está bem estabelecida e o tratamento dessa condição é realizado de forma rotineira, sendo considerado uma opção segura, viável e com resultados comprovados a curto e a médio prazo.^24,25^

O implante percutâneo transeptal mitral em próteses biológicas disfuncionais, por sua vez, evoluiu significativamente nos últimos anos com ajustes técnicos importantes, aumentando a taxa de sucesso e a segurança e reduzindo o tempo do procedimento.^18^ Inicialmente, foram descritas técnicas de implante transeptal utilizando uma punção transapical para auxiliar o deslocamento e o posicionamento da prótese (*apical rail*), porém a manipulação ventricular e a necessidade de toracotomia resultaram em maiores taxas de complicações hemorrágicas.^26^ Em 2015, Coylewright et al.,^19^ publicaram uma série de quatro pacientes submetidos ao tratamento de disfunção de bioprótese ou anel cirúrgico mitral, utilizando somente o acesso venoso transfemoral, sem *apical rail*.^19^ Com a técnica transeptal, evita-se a manipulação do ápice do ventrículo esquerdo e restringe-se o acesso vascular à veia femoral, o que resulta em taxas de complicações vasculares inferiores àquelas encontradas em procedimentos que utilizam a artéria femoral, como é o caso do implante transcateter de bioprótese valvar aórtica. O procedimento realizado por acesso venoso transeptal tornou-se mais simples e com resultados previsíveis, podendo ser aplicado de forma mais segura em pacientes considerados de alto risco cirúrgico.

Na presente série, foi possível realizar o implante da bioprótese transcateter em 100% dos casos, obteve-se sucesso do procedimento em 88,2%, sendo a taxa de sobrevida aos 30 dias de 94,1%. Esses resultados muito se assemelham aos encontrados na literatura. Em 2016, Eleid et al.,^18^ publicaram uma série com 33 procedimentos *valve-in-valve* (VIV). O implante da prótese foi realizado por via transfemoral em todos os casos, a taxa de sucesso do procedimento foi 93,9% e a sobrevida em 30 dias foi de 88,9%.^18^ Publicação posterior demonstrou que a melhora clínica e o funcionamento da prótese foram sustentados ao longo de um ano de seguimento.^14^ Recentemente, Yoon et al.,^21^ publicaram um registro multicêntrico internacional com um total de 521 pacientes, dos quais 322 foram submetidos ao tratamento VIV, 141 foram submetidos ao tratamento *valve-in-ring* e 58 foram submetidos ao tratamento *valve-in-mitral annular calcification* (MAC). A via de acesso mais utilizada foi a transeptal (59,5%), seguida pela transapical (39,5%), com poucos casos de via transatrial (1%). Nesse estudo, a taxa de sucesso do procedimento foi de 73,6% e a sobrevida em 30 dias de 93,8%, considerando apenas o grupo VIV.^21^

Apesar da evolução técnica e do aumento da experiência mundial com o procedimento nos últimos anos, estudos comparativos entre o tratamento cirúrgico tradicional (retroca valvar mitral) e o tratamento transcateter VIV ainda são escassos. Kamioka et al.,^10^ em 2018, publicaram um estudo multicêntrico, retrospectivo, comparando implante transcateter VIV (62 pacientes) com retroca valvar cirúrgica mitral (59 pacientes). No grupo de intervenção transcateter, 77% dos pacientes foram tratados por via transeptal, apresentaram menor taxa de sangramento maior, menor taxa de arritmias atriais e menor tempo de internação. Embora houvesse diferença estatisticamente significativa na idade média e no risco cirúrgico entre os grupos, a mortalidade em 30 dias foi semelhante (transcateter VIV 3,2%; retroca cirúrgica 3,4%), assim como o gradiente médio residual (transcateter VIV 7,1 mmHg; retroca cirúrgica 6,5 mmHg) e a presença de regurgitação mitral moderada ou importante após a intervenção (transcateter VIV 3,8%; retroca cirúrgica 5,6%). No seguimento de um ano, a taxa de mortalidade foi similar nos dois grupos (transcateter VIV 11,3%; retroca cirúrgica 11,9%). Estudos clínicos randomizados considerando os diversos estratos de risco da intervenção em cada paciente são necessários para compreendermos melhor o papel do procedimento transcateter no tratamento da disfunção de bioprótese cirúrgica em posição mitral.^10^

Cabe destacar, entretanto, que o sucesso do procedimento depende de um adequado planejamento prévio, incluindo uma criteriosa avaliação clínica considerando o *status* global do paciente e sua capacidade funcional, assim como o estudo pormenorizado das alterações estruturais já estabelecidas durante a evolução da valvopatia. A avaliação de risco cirúrgico é primordial e deve ser realizada aplicando os escores de risco tradicionais, mas também de forma individualizada, considerando outras comorbidades não contempladas por tais escores e a experiência prévia da equipe do *valve team*. A avaliação ecocardiográfica detalhada das características da disfunção valvar e suas repercussões, bem como a presença de trombo nas cavidades cardíacas, especialmente no átrio esquerdo, é fundamental. Outro passo importante no planejamento é a escolha do tamanho e do modelo da prótese a ser implantada, levando em conta as especificações técnicas dos dispositivos previamente implantados e as medidas realizadas através do ecocardiograma e da tomografia computadorizada.

Parte essencial do planejamento pré-operatório é a análise do risco de obstrução da VSVE após o implante valvar transcateter. Alguns dos principais preditores dessa complicação são o tamanho da VSVE, o tamanho da cavidade ventricular esquerda, o ângulo mitro-aórtico e a distância entre o ânulo mitral e o septo interventricular.^15,17^ Esses dados podem ser avaliados inicialmente pelo ecocardiograma; entretanto, a tomografia apresenta fundamental importância já que alguns parâmetros só podem ser avaliados por esse método. Em estudo recente, Yoon et al.,^17^ avaliaram os preditores angiotomográficos dessa temível complicação e demonstraram que a distância entre o ânulo mitral e o septo interventricular e a área estimada da VSVE após implante apresentam melhor correlação com o desenvolvimento de obstrução de VSVE do que o ângulo mitro-aórtico.^17^ Em nossa série, a obstrução da VSVE ocorreu em 2 casos (11,7%), sendo que, em 1 deles, resultou no óbito do paciente poucas horas após a intervenção.

Outro aspecto técnico importante para a segurança do procedimento é guiar a punção transeptal por ecocardiograma transesofágico, no sentido de minimizar a ocorrência de complicações hemorrágicas. Há ainda a necessidade de dilatar o septo interatrial com balão para a passagem da prótese, o que resulta em uma comunicação interatrial residual, em geral sem repercussão hemodinâmica. O posicionamento da prótese a ser implantada deve ser feito com precisão e é, em geral, guiado por marca radiopaca da bioprótese cirúrgica disfuncional. No entanto, como algumas das próteses cirúrgicas utilizadas para tratamento das valvopatias mitrais não apresentam marca radiopaca, torna-se fundamental o emprego do ecocardiograma tridimensional para obter melhor posição e alinhamento da prótese.

### Limitações do estudo

Trata-se de um estudo retrospectivo, multicêntrico, descrevendo a experiência inicial de 12 hospitais em 6 estados brasileiros. Apesar de esta série incluir grande parte dos procedimentos TMVIV-via transeptal do país, o pequeno número de casos ainda é uma limitação. Uma amostra maior de pacientes com maior tempo de seguimento e a comparação com as modalidades cirúrgica e transapical fazem-se necessárias para o conhecimento da real utilidade dessa nova modalidade de tratamento em nosso meio.

## Conclusão

A primeira experiência brasileira de TMVIV-via transeptal demonstra a segurança, a efetividade e a significativa melhora funcional de pacientes tratados por essa nova técnica. A obstrução da VSVE é uma complicação potencialmente fatal, reforçando a importância da seleção dos pacientes e do planejamento do procedimento.
