# Supplementary material for: Implante Percutâneo Transeptal de Bioprótese em Disfunção de Prótese Valvar Cirúrgica Mitral – Experiência Multicêntrica Brasileira
Source: Arq Bras Cardiol. 2020 Sep 18;115(3):515–24. [Article in Portuguese] doi: 10.36660/abc.20190252 (PMC9363107; doi:10.36660/abc.20190252)
Supplement: Translation

# Translation: Percutaneous Transseptal Bioprosthetic Implantation in Failed Prosthetic Surgical Mitral Valve – Brazilian Multicenter Experience

**Authors:** Pedro Felipe Gomes Nicz, Pedro Henrique M. Craveiro de Melo, Pedro Henrique Ferro de Brito, Eliane Nogueira Lima, Ricardo Cavalcante e Silva, Maurício Lopes Prudente, Fernando Henrique Fernandes, Maurilio Onofre Deininger, Marcelo Antônio Cartaxo Queiroga Lopes, Fúlvio Soares Petrucci, Fernando Roquette Reis, Marcos Antonio Marino, Rodrigo de Castro Bernardes, Eduardo Pessoa de Melo, Marco Antonio Praça Oliveira, José Armando Mangione, Fernanda Marinho Mangione, Carlos Henrique Eiras Falcão, Estêvão Carvalho de Campos Martins, Walter Lunardi, Fernando Bacal, Flávio Tarasoutchi, Fábio Sândoli de Brito

**Affiliations:** 1 Brazil Universidade de São Paulo Faculdade de Medicina Hospital das Clínicas Instituto do Coração – Hemodinâmica, São Paulo, SP - Brazil; 2 Brazil Hospital São Camilo Pompeia - Cardiologia Intervencionista, São Paulo, SP - Brazil; 3 Brazil Hospital Sírio-Libanês, São Paulo, SP - Brazil; 4 Brazil Faculdade de Ciências Médicas da Santa Casa de São Paulo, São Paulo, SP - Brazil; 5 Brazil Hospital Encore, Goiânia, GO - Brazil; 6 Brazil Hospital Alberto Urquiza Wanderley, João Pessoa, PB - Brazil; 7 Brazil Hospital Memorial São Francisco, João Pessoa, PB - Brazil; 8 Brazil Hospital Madre Teresa, Belo Horizonte, MG - Brazil; 9 Brazil Hospital Esperança Olinda - Rede D’Or São Luiz, Olinda, PE - Brazil; 10 Brazil Hospital Beneficência Portuguesa de São Paulo, São Paulo, SP- Brazil; 11 Brazil Complexo Hospitalar de Niterói, Niterói, RJ - Brazil; 12 Brazil Hospital de Força Aérea do Galeão, Rio de Janeiro, RJ - Brazil; 13 Brazil Universidade de São Paulo Instituto do Coração, São Paulo, SP - Brazil; 14 Brazil Universidade de São Paulo Faculdade de Medicina Hospital das Clínicas Instituto do Coração – Cardiologia, São Paulo, SP – Brazil

**Keywords:** Mitral Valve Stenosis/surgery, Transcatheter Mitral Valve Replacement, Bioprosthesis, Echocardiography, Transesophageal/methods, Heart Valve Prosthesis Implantation/trends

## Abstract

**Background:**

Percutaneous intervention in patients with bioprosthetic mitral valve dysfunction is an alternative to conventional surgical treatment.

**Objectives:**

To report the first Brazilian experience with transseptal transcatheter bioprosthetic mitral valve-in-valve implantation (transseptal-TMVIV).

**Methods:**

Patients with surgical bioprosthetic dysfunction submitted to transseptal-TMVIV in 12 Brazilian hospitals were included. The significance level adopted was p<0.05.

**Results:**

From June/2016 to February/2019, 17 patients underwent transseptal-TMVIV. Their median age was 77 years (IQR,70-82) and median Society of Thoracic Surgeons predicted risk of mortality (STS-PROM) score was 8.7% (IQR,7.2-17.8). All patients had limiting symptoms of heart failure (FC≥III) and 5 (29.4%) had undergone more than one previous thoracotomy. Transseptal-TMVIV was successful in all patients. Echocardiographic assessment showed a significant reduction in mean mitral valve gradient (pre-intervention, 12±3.8 mmHg; post-intervention, 5.3±2.6 mmHg; p<0.001), in addition to an increase in mitral valve area (pre-intervention, 1.06±0.59 cm^2^; post-intervention, 2.18±0.36 cm^2^; p<0.001) sustained for 30 days. There was a significant and immediate reduction in the pulmonary artery systolic pressure, with an additional reduction in 30 days (pre-intervention, 68.9±16.4 mmHg; post-intervention, 57.7±16.5 mmHg; 30 days, 50.9±18.7 mmHg; p<0.001). During follow-up (median, 162 days; IQR, 102-411), significant clinical improvement (FC≤II) was observed in 87.5% of the patients. One patient (5.9%) had left ventricular outflow tract (LVOT) obstruction and died right after the procedure, and another died at 161 days of follow-up.

**Conclusion:**

The first Brazilian experience with transseptal-TMVIV shows the safety and effectivity of the new technique. The LVOT obstruction is a potentially fatal complication, reinforcing the importance of patients’ selection and of procedural planning. (Arq Bras Cardiol. 2020; 115(3):515-524)

## Introduction

Mitral valve surgical replacement and repair are the treatment of choice for a large number of primary mitral valve impairments; however, prosthetic valve degeneration and consequent failure jeopardize the durability of long-term therapy.^1-3^ Up to 35% of the patients submitted to mitral valve surgical treatment need a new intervention after a median period of 8 years, with an in-hospital mortality rate ranging from 8% to 12% and a mean length of hospital stay of 17 days.^4-8^ To some patients with prosthetic mitral valve dysfunction and indication for surgical intervention, especially those with multiple comorbidities and previous surgical manipulations, surgery is contraindicated because of the high risk associated with the procedure. In such situations, transcatheter intervention is an alternative to surgical treatment.

To the transcatheter intervention, the transapical access, already performed in Brazil, is advantageous because it provides direct access to the mitral valve from the cardiac apex, in addition to its lower morbidity as compared to that of the conventional surgery.^9,10^ However, it is associated with high rates of hemorrhagic complications, need for thoracic drainage because of pleural cavity opening, and, thus, prolonged length of hospital stay.^11-13^

Aiming at reducing the procedure risk and the length of hospital stay, the technique of transseptal transcatheter bioprosthetic mitral valve-in-valve implantation (transseptal-TMVIV) has been developed. Initial reports and case series have shown that the technique is feasible and safe, with good clinical results in the mid-term follow-up.^14^

This study reports the first Brazilian experience with transseptal-TMVIV to treat patients with bioprosthetic mitral valve failure at high risk for a new surgical intervention.

## Methods

This study included patients with significant dysfunction of a surgical bioprosthetic mitral valve (stenosis, insufficiency, or both) submitted to transseptal-TMVIV in 12 Brazilian hospitals. When this case series was conducted, that was an off-label procedure, despite being widely performed in many countries worldwide. More recently, transseptal-TMVIV has been approved in Brazil.

All patients were assessed by the local heart team and classified as at high risk for surgery. Demographic and clinical data, in addition to data from the complementary tests and the procedure were collected. Clinical follow-up abided by the local medical practice, as did the indication for the post-intervention use of antiplatelets and/or anticoagulants.

### Preprocedural planning

All patients underwent preprocedural transesophageal echocardiography to document and quantify heart valve dysfunction, and to assess the mechanism of prosthetic valve failure, the presence of paravalvular regurgitation and the suggestive signs of infective endocarditis.

Preprocedural coronary computed tomographic angiography enabled the assessment of the real inner diameter of the dysfunctional prosthetic valve, its angulation, and its relationship with left ventricular outflow tract (LVOT), as shown in Figure 1.^15-17^ When available, the previous surgery report was useful to provide information on the type and technical specifications of the prostheses implanted.

**Figure 1:** – Preprocedural planning - Cardiac computed tomography.15-17

### Procedures

The procedures were performed in a traditional catheterization laboratory or hybrid endovascular suite, with the patient under general anesthesia and the aid of intraoperative transesophageal echocardiography. The left femoral artery was accessed with a 5F introducer for invasive monitoring of blood pressure and occasional left ventriculography. The left femoral vein was used to position the transvenous temporary pacemaker lead. The right femoral vein was used for the transseptal puncture and transcatheter implantation of the prosthesis. A Perclose ProGlide™ device (Abbott Vascular Devices, Santa Clara, California, USA) was prepositioned for further venous hemostasis.

During the procedure, the interatrial septum was punctured under echocardiographic guidance, using the usual techniques. To facilitate the manipulation of the devices in the left atrium and mitral valve crossing, septal puncture in the posterior and inferior or mid septal regions was chosen.

Heparin was administered aiming at achieving an activated clotting time longer than 300 seconds. After septal puncture, by use of a 0.035” exchange guidewire in the left superior pulmonary vein, an 8F Agilis NXT steerable introducer (St Jude Medical, St Paul, MN, USA) was advanced up to the left atrium. A 5F JR diagnostic catheter was used to cross the mitral valve by passing a hydrophilic wire (straight tip), guided by transesophageal echocardiography and fluoroscopy, perpendicular to the mitral valve ring. Then, one or two Safari guidewires (Boston Scientific, MN, USA) or Amplatz Extra-Stiff (Cook Medical, Bloomington, Indiana, USA) wire guides were positioned in the left ventricle. For the first patient of the case series, the arterial loop technique was chosen, catching and exteriorizing the 0,035” guidewire via femoral arterial access to facilitate navigation and positioning of the prosthesis.

Balloon-expandable prostheses SAPIEN XT and SAPIEN 3 (Edwards LifeSciences, Irvine, California, USA) were chosen because of characteristics, such as low profile, flexibility, radial force, previous experience in valve-in-valve implantations, in addition to good performance in international reports and case series.^15,18-22^ The size of the prosthesis was chosen according to tomographic angiographic assessment and recommendations from the surgical prosthesis manufacturer, aiming at a 5-10% oversizing. The prosthesis was positioned in the balloon-catheter in the direction used for anterograde implantation, similarly to the preparation for transapical aortic implantation.

The introducer specific for the SAPIEN 3 or SAPIEN XT prosthesis was positioned and the interatrial septum was dilated with inflation of 12-16-mm diameter and 40-mm length balloons.

After septal dilation, the prosthesis was inserted in its release system in the inferior vena cava, where it was adjusted and aligned with the balloon. With the aid of the flexion system of the release device, the prosthesis was advanced through the septum and positioned in the mitral valve topography. Then, guided by transesophageal echocardiography and fluoroscopy, the device was positioned in the ideal place for release. With the prosthesis positioned, the pacemaker was set at the rate of 180 bpm (rapid pacing), and the release balloon was inflated slowly, with small positioning adjustments, as required, aiming at achieving the prosthesis final ‘10-20% atrial and 80-90% ventricular’ position, with the ventricular face more expanded than the atrial face to minimize the likelihood of atrial embolism.

After the release of the prosthesis, a detailed assessment of mitral valve implantation and functioning was performed and repeated after withdrawal of the release system from the left cavities. Then, the residual interatrial communication was assessed and quantified. At the end of the procedure, left ventriculography was performed to assess the position, the presence of mitral regurgitation and possible procedure-related complications. The effect of heparin was reversed, and the femoral introducer was removed with the aid of a Perclose ProGlide™ device (Abbott Vascular Devices, Santa Clara, California, USA) previously positioned to provide hemostasis. The arterial introducer and the temporary pacemaker were withdrawn right after the procedure. Figure 2 illustrates the steps of the procedure.

**Figure 2:** – Steps of the transseptal transcatheter bioprosthetic mitral valve-in-valve implantation.

### Statistical analysis

All continuous variables were tested for normality using the Kolmogorov–Smirnov or Shapiro-Wilk tests, both corroborating the results. The continuous variables with normal distribution were presented as mean and standard deviation, and those without normal distribution, as median and interquartile range (IQR). The categorical variables were presented as frequencies (number and percentage). For the sequential analysis of the continuous variables in the same patient, the generalized estimation equations (GEE) method was used with Bonferroni multiple comparisons. To analyze the evolution of heart failure functional class (FC), Wilcoxon paired test was used. The SPSS for Windows, version 22.0, was used for the statistical analyses. The p values < 0.05 were considered statistically significant.

### Ethical considerations

The formal written consents related to the procedure were obtained before each intervention. This study was submitted to and approved by the Ethics Committee in Research and was conducted according to Resolution 466/12 and the Brazilian Health Council complements.

## Results

From June 2016 to January 2019, 17 patients underwent transseptal-TMVIV always conducted by an experienced professional acquainted with the technique (FSBJ). Table 1 shows the demographic and baseline clinical characteristics. The patients’ ages ranged from 29 to 85 years, with a median of 77 years (IQR, 70-82). Eleven patients (64.7%) were of the female sex, and 9 (52.9%) had atrial fibrillation. The most frequent etiologies of primary mitral valve dysfunction were myxomatous degeneration and rheumatic fever impairment, occurring in 7 patients (41.2%). The median Society of Thoracic Surgeons predicted risk of mortality (STS-PROM) score was 8.7% (IQR, 7.2-17.8%). Five patients (29.4%) had undergone more than one previous cardiac surgery, with a median time since the last mitral valve replacement of 9 years (IQR, 8-10). All patients had significant functional limitation (heart failure FC III or IV) and significant pulmonary artery hypertension, with median pulmonary artery systolic pressure (PASP) of 69.5 mmHg (IQR, 57.3-73). The median left ventricular ejection fraction (LVEF) was 63% (IQR, 57-65%), and 2 patients had important left ventricular dysfunction with LVEF lower than 35%.

**Table 1:** – Demographic and baseline clinical characteristics

	(n = 17)
Age, years	73.8 ± 13.0
Female sex, n (%)	11 (64.7)
NYHA functional class III or IV, n (%)	17 (100)
NYHA functional class IV, n (%)	9 (52.9)
STS score, %	6.2 ± 16.0
Previous heart surgeries (n≥ 2 surgeries), n (%)	5 (29.4)
Diabetes mellitus, n (%)	5 (29.4)
Creatinine, mg/dL	1.3 ± 0.7
Arterial hypertension, n (%)	8 (47.0)
Atrial fibrillation, n (%)	9 (52.9)
Previous stroke, n (%)	4 (23.5)
Chronic pulmonary disease, n (%)	9 (52.9)
Previous AMI, n (%)	4 (23.5)
Previous CABG, n (%)	4 (23.5)
Previous coronary angioplasty, n (%)	3 (17.6)
Echocardiographic data	
Mean mitral valve gradient, mmHg	10.6 ± 5.4
PASP, mmHg	68.9 ±16.4
LVEF, %	59.3 ± 13.0
Mechanism of failure	
Regurgitation, n (%)	3 (17.6)
Stenosis, n (%)	10 (58.8)
Combined, n (%)	4 (23.5)

*NYHA: New York Heart Association; STS: Society of Thoracic Surgeons; AMI: acute myocardial infarction; CABG: coronary artery bypass grafting; PASP: pulmonary artery systolic pressure; LVEF: left ventricular ejection fraction.*

The most common failure mechanism was pure prosthetic mitral stenosis, present in 10 (58.8%) patients. Four other patients (23.5%) had combined dysfunction, and 3 (17.6%) had pure mitral insufficiency (Table 2). Tomographic angiographic assessment was performed in 15 patients (88.2%), indicating that the median angle between the mitral and aortic planes was 123^o^ (IQR, 117-134), and, in 35.3% of the patients, that angle was lower than 120^o^.

**Table 2:** – Hemodynamic and valvular characteristics per patient

Patient #	Previous prosthesis	Prosthesis’ age (years)	Dysfunction type	Size (mm)	SAPIEN model	SAPIEN size (mm)	Mean gradient (mmHg)		Regurgitation degree		PASP (mmHg)	
							pre	post	pre	post	pre	post (30 days)
1	Biocardio	8	Stenosis	27	XT	26	18	8	0	0	90	87
2	St. Jude Biocor	3	Stenosis	29	XT	26	16	12	0	0	82	72
3	Braile	8	Stenosis	31	S3	29	12	-	1	0	69	-
4	Undetermined	3	Combined	29	S3	29	7	3	4	0	65	-
5	Undetermined	10	Combined	29	XT	26	9	6	4	1	105	75
6	St. Jude Biocor	12	Stenosis	29	XT	26	10	4	0	0	87	-
7	St. Jude Biocor	14	Insufficiency	31	XT	29	-	9	4	0	53	40
8	Undetermined	6	Stenosis	29	XT	26	13	3	0	1	-	30
9	Undetermined	13	Stenosis	25	S3	26	20	5	1	0	70	-
10	Carpentier-Edwards	9	Stenosis	29	S3	29	14	4	1	0	55	38
11	Carpentier-Edwards	8	Stenosis	29	S3	29	12	5	1	0	68	51
12	Carpentier-Edwards Magna	12	Combined	29	S3	29	10	4	4	0	70	57
13	Carpentier-Edwards	10	Stenosis	29	S3	29	8	3	1	0	40	36
14	Carpentier-Edwards Magna	10	Stenosis	29	S3	29	13	3	1	0	50	55
15	Braile	10	Combined	27	S3	26	11	6	2	1	58	38
16	Labcor	8	Insufficiency	31	S3	29	-	4	4	0	70	-
17	Cardioprotese	1	Insufficiency	29	S3	26	7	-	4	0	70	32

*PASP: pulmonary artery systolic pressure.*

Positioning and implantation were performed as planned, via transseptal access, in all patients. In five patients, the dysfunctional prosthetic mitral valve showed no radiopaque mark that could aid percutaneous valvular implantation, thus, their transcatheter prosthetic valve positioning was guided by three-dimensional transesophageal echocardiography.

The medians of procedural and fluoroscopy durations and contrast volume were 125 minutes (IQR, 100-148), 25 minutes (IQR, 22-40), and 50 mL (IQR, 43-141mL), respectively. SAPIEN XT and SAPIEN 3 balloon-expandable transcatheter prostheses were used in 6 patients (35.3%) and 11 patients (64.7%), respectively. The 29-mm prosthesis was chosen for 52.9% of the patients, while the 26-mm prosthesis, for the others (47.1%) (Table 2).

Although prosthetic positioning and release occurred as planned, one patient (5.9%) had LVOT obstruction, detected on echocardiography and confirmed by use of direct measurement of the intraventricular gradient, followed by rapid hemodynamic deterioration, refractory cardiogenic shock and death hours after the procedure. Because of that patient’s extreme baseline clinical severity, tomographic angiography could not be performed before the intervention. In another patient, a 20-mmHg invasive gradient in the LVOT was detected right after the procedure with no clinical repercussion. No other procedure-related complications, such as need for conversion into emergency surgery, acute myocardial infarction, stroke, major vascular complications, and major bleedings, were observed. Within 30 days, no patient had prosthetic thrombosis, stroke or need for new cardiac interventions. The median length of hospital stay was 7 days (IQR, 4-14).

During follow-up, whose median duration was 162 days (IQR, 102-411), the patients of this case series had favorable clinical course, and a significant improvement in FC (≤ II) was observed in 14 patients (87.5%) (Figure 3). Of the 16 patients discharged, 4 (25%) were readmitted due to heart failure. One patient, who remained in FC IV during follow-up with severe pulmonary hypertension and multiple readmissions, died 161 days after the intervention. The procedural success rate, according to the definitions established by the Mitral Valve Academic Research Consortium (MVARC),23 was 88.2%.

**Figure 3:** – Functional class - baseline vs. follow-up.

The echocardiographic assessment showed a significant reduction in the mean mitral valve gradient, sustained for 30 days and associated with the increase in the valve area (Figure 4). In addition, a significant reduction in PASP was observed immediately after the procedure, with an additional reduction after 30 days of follow-up (Figure 4). No mitral regurgitation greater than mild (>1+) was detected after the implantations, and no patient showed hemodynamically significant interatrial communication that required intervention immediately or during follow-up.

**Figure 4:** – Echocardiographic data - pre-intervention, post-intervention, at 30 days of follow-up. PASP: pulmonary artery systolic pressure; MG: mitral valve gradient; MVA: mitral valve area

## Discussion

This case series about the initial experience with transseptal-TMVIV in surgical prosthetic mitral valve dysfunction shows that the procedure is safe and effective, with results similar to those reported in international series, and thus should be considered an alternative to the traditional surgical treatment of high-risk patients.

The percutaneous approach to bioprosthetic aortic valve dysfunction has been well established and routinely performed to treat that condition, being considered a safe and feasible option, with proven results in the short and intermediate run.^24,25^

Percutaneous transseptal valve implantation in dysfunctional bioprosthetic mitral valves has significantly evolved in past years with important technical adjustments, which increased the procedural success rate and safety and reduced its duration.18 Initially, transseptal implantation techniques using a transapical puncture to aid the prosthetic displacement and positioning (apical rail) have been described; however, the ventricular manipulation and need for thoracotomy have resulted in higher rates of hemorrhagic complications.^26^ In 2015, Coylewright et al.^19^ published a case series of four patients submitted to the treatment of degenerative mitral bioprosthetic valves or rings, by using only femoral venous access, with no apical rail.^19^ The transseptal technique prevents the manipulation of the left ventricular apex, limiting the vascular access to the femoral vein, which results in rates of vascular complications lower than those found in procedures using the femoral artery, such as transcatheter implantation of bioprosthetic aortic valves. The procedure performed via transseptal venous access has become simpler, with predictable results and can be applied more safely to patients at high risk for surgery.

In the present case series, transcatheter implantation of the bioprosthetic valve could be performed in 100% of the patients, with procedural success rate of 88.2% and 30-day survival of 94.1%. These results are very similar to those found in the literature. In 2016, Eleid et al.^18^ published a case series with 33 valve-in-valve (VIV) procedures, the prosthesis being implanted via transfemoral access in all cases, with procedural success rate of 93.9% and 30-day survival of 88.9%.^18^ Another study has shown clinical improvement and prosthetic valve functioning sustained over an one-year follow-up.^14^ Recently, Yoon et al.^21^ have published an international multicenter registry with 521 patients, 322 of whom underwent VIV treatment, 141 underwent valve-in-ring treatment, and 58 underwent valve-in-mitral annular calcification treatment. Transseptal access was the most used (59.5%), followed by transapical (39.5%) and transatrial (1%) accesses. In that study, the procedural success rate was 73.6% and the 30-day survival was 93.8%, considering only the VIV group.21

Despite improvements in the technique and growing experience with the procedure across countries in recent years, studies comparing the traditional surgical treatment (redo surgical mitral valve replacement) and the transcatheter VIV treatment are scarce. In 2018, Kamioka et al.^10^ published a multicenter retrospective study comparing transcatheter VIV implantation (62 patients) and redo surgical mitral valve replacement (59 patients). In the transcatheter intervention group, 77% of the patients were approached via transseptal access, had a lower major bleeding rate, lower atrial arrhythmia rate and shorter length of hospital stay. Despite the statistically significant difference in mean age and in surgical risk between the groups, mortality at 30 days was similar (transcatheter VIV, 3.2%; surgical redo, 3.4%), as were the residual mean mitral valve pressure (transcatheter VIV, 7.1 mmHg; surgical redo, 6.5 mmHg) and the presence of moderate or important mitral regurgitation (transcatheter VIV 3.8%; surgical redo, 5.6%) after the intervention. The 1-year mortality rate was similar in both groups (transcatheter VIV, 11.3%; surgical redo, 11.9%). Randomized clinical trials considering the different risk levels of the intervention for each patient are required to better clarify the role of the transcatheter procedure to treat the dysfunction of surgical bioprosthetic mitral valves.^10^

It is worth noting, however, that procedural success depends on previous proper planning, such as careful clinical assessment considering the patient’s overall *status* and functional capacity, in addition to a thorough study of the structural alterations established during the progression of valvulopathy. Assessing the surgical risk is essential and should be performed by applying traditional risk scores, but in an individualized way, considering the previous experience of the valve team and other comorbidities not contemplated in those scores. The detailed echocardiographic assessment of the characteristics of valvular dysfunction and its repercussions, in addition to the presence of an intracavitary thrombus, especially in the left atrium, is fundamental. Another important step in planning is choosing the size and model of the prosthesis to be implanted, considering not only the technical specifications of the devices previously implanted, but the echocardiographic and computed-tomographic measurements as well.

An essential part of the preoperative planning is the analysis of the risk of LVOT obstruction after transcatheter valvular implantation. The major predictors of that complication are the sizes of the LVOT and of the left ventricular cavity, the mitral-aortic angle, and the distance between the mitral ring and the interventricular septum.^15,17^ Those data can be initially assessed on echocardiography; however, some parameters can only be assessed by using computed tomography, which, therefore, is of fundamental importance. In a recent study, Yoon et al.^17^ have assessed the computed-tomographic predictors of LVOT obstruction and have shown that the distance between the mitral ring and the interventricular septum, as well as the estimated LVOT area after implantation, correlates better with the development of LVOT obstruction than the mitral-aortic angle does.^17^ In our case series, LVOT obstruction was observed in two patients (11.7%), and one of them died a few hours after the intervention.

Another important technical aspect for procedural safety is to guide the transseptal puncture with transesophageal echocardiography to minimize the occurrence of hemorrhagic complications. In addition, the interatrial septum requires balloon-dilatation to pass the prosthesis, which results in a residual interatrial communication, usually with no hemodynamic repercussion. The positioning of the prosthesis to be implanted should be precise, being usually guided by a radiopaque mark of the failed surgical bioprosthesis. However, because some of the surgical prostheses used to treat mitral valvulopathies have no radiopaque mark, the use of three-dimensional echocardiography is fundamental to better position and align the prosthesis.

### Study limitations

This is a retrospective multicenter study, describing the initial experience of 12 hospitals in 6 Brazilian states. Although this case series includes a great part of the transseptal-TMVIV procedures performed in Brazil, its small number of cases is a limitation. A larger sample size with a longer follow-up, as well as the comparison with the surgical and transapical modalities, is necessary to know the real usefulness of that new modality of treatment in our country.

### Conclusion

The first Brazilian experience with transseptal-TMVIV shows the safety and effectivity of the new technique and the significant functional improvement of patients treated with it. The LVOT obstruction is a potentially fatal complication, reinforcing the importance of patients’ selection and of procedural planning.
